# The moderating effect of diet on the relationship between depression and Alzheimer’s disease‐related blood‐based biomarker

**DOI:** 10.1002/alz.091148

**Published:** 2025-01-09

**Authors:** Hilal Salim Al Shamsi, Samantha L Gardener, Stephanie Rainey Smith, Hamid R. Sohrabi, Warnakulasuriya Mary Ann Dipika Binosha Fernando, Ralph N Martins

**Affiliations:** ^1^ ECU university, Joondalup, Western Australia Australia; ^2^ Murdoch University, Perth, Western Australia Australia; ^3^ Edith Cowan University, Perth, Western Australia Australia; ^4^ Alzheimer's Research Australia, Perth, Western Australia Australia; ^5^ Edith Cowan University, Joondalup, Western Australia Australia; ^6^ Centre for Healthy Ageing, Murdoch University, Murdoch, Western Australia Australia; ^7^ Centre of Excellence for Alzheimer’s Disease Research and Care, School of Medical and Health Sciences, Edith Cowan University, Joondalup, Western Australia Australia; ^8^ Australian Alzheimer's Research Foundation, Nedlands, Western Australia Australia; ^9^ School of Medical and Health Sciences, Edith Cowan University, Joondalup, Western Australia Australia; ^10^ Ageing, Cognition and Exercise Research Group, Murdoch Australia; ^11^ Australian Alzheimer’s Research Foundation, Perth, Western Australia Australia; ^12^ Murdoch University, Murdoch, Western Australia Australia; ^13^ School of Psychological Science, University of Western Australia, Crawley, Western Australia Australia; ^14^ Department of Biomedical Sciences, Macquarie University, Sydney, NSW Australia; ^15^ Centre for Healthy Ageing, Murdoch University, Murdoch, Perth, Western Australia Australia; ^16^ Edith Cowan University, Perth Australia; ^17^ McCusker Alzheimer’s Research Foundation, Perth, Western Australia Australia; ^18^ Macquarie University, Sydney, NSW Australia; ^19^ Centre for Ageing, Cognition and Wellbeing, Macquarie University, North Ryde, NSW Australia

## Abstract

**Background:**

Interplay between diet, depression, and Alzheimer's disease (AD) shows promise as an important area of study for early intervention and therapy. Associations between both depression and diet and heightened AD risk underscore the need to examine the moderating effect of dietary patterns on the relationship between depression, and AD‐related blood‐based biomarkers.

**Method:**

Data from cognitively unimpaired older adults (n=89; age ≥60 years) enrolled in the Australian Imaging, Biomarkers and Lifestyle (AIBL) study were included. Participants completed a food frequency questionnaire, self‐report measures of depression, and had AD‐related blood‐based biomarker levels for phosphorylated tau (ptau181) 181, Aβ40, Aβ42, Neurofilament Light Chain (NfL), and Glial Fibrillary Acidic Protein (GFAP). Scores for two dietary patterns, 1) Mediterranean diet (MeDi), and 2) western diet were generated for each individual. Moderation and simple slope analyses were used to examine the interactions between dietary patterns, depression, and biomarker levels.

**Result:**

Depression was positively associated with NfL levels in males with lower than mean MeDi score adherence (β=3.759, SE=1.248, p=0.005). Depression was also positively associated with NfL levels in Apolipoprotein E (APOE) ε4 allele non‐carriers with lower than mean and mean MeDi score adherence (β=7.384, SE=1.972, p<0.001; β=3.797, SE=1.004, p<0.001, respectively).

Moreover, depression was positively associated with Aβ40 levels in APOE ε4 allele non‐carriers with lower than mean and mean MeDi score adherence (β=16.837, SE=5.242, p=0.002; β=7.502, SE=2.669, p=0.005, respectively).

No moderating effects of MeDi on the relationship between depression and levels of ptau181, Aβ42, and GFAP were observed. Furthermore, western diet had no moderating effect on the relationship between depression, and AD‐related blood‐based biomarkers.

**Conclusion:**

This study highlights MeDi adherence as a potential moderator of the relationship between mood and AD‐related blood‐based biomarker levels. These findings also emphasize the importance of gender‐ and genotype‐specific approaches to depression‐cognition‐diet research and highlight the need for further investigation.

